# Early Hypertransaminasemia after Kidney Transplantation: Significance and Evolution According to Donor Type

**DOI:** 10.3390/jcm10215168

**Published:** 2021-11-04

**Authors:** Eulàlia Solà-Porta, Dolores Redondo-Pachón, Carlos Arias-Cabrales, Diego Navazo, Anna Buxeda, Carla Burballa, Marta Crespo, Montserrat García-Retortillo, Julio Pascual, María José Pérez-Sáez

**Affiliations:** 1 Nephrology Department, Hospital del Mar, 08003 Barcelona, Spain; esola@psmar.cat (E.S.-P.); mredondopachon@psmar.cat (D.R.-P.); cariascabrales@psmar.cat (C.A.-C.); dnavazo90@gmail.com (D.N.); abuxeda@psmar.cat (A.B.); cburballa@psmar.cat (C.B.); mcrespo@psma.cat (M.C.); julpascual@gmail.com (J.P.); 2 Liver Section, Hospital del Mar, 08003 Barcelona, Spain; mgarciaretortillo@psmar.cat

**Keywords:** donor after circulatory death, early, hypertransaminasemia, kidney transplantation, transaminases

## Abstract

Early hypertransaminasemia after kidney transplantation (KT) is frequent. It has been associated with the crosstalk produced between the liver and the kidney in ischemia-reperfusion situations. However, the influence of the donor type has not been evaluated. We present a retrospective study analyzing the increase in serum aspartate aminotransferase/alanine aminotransferase (AST/ALT) during the first three months post-KT in 151 recipients who received thymoglobulin as induction therapy, either from brain-death donors (DBD, *n* = 75), controlled circulatory death donors (cDCD, *n* = 33), or uncontrolled DCD (uDCD, *n* = 43). Eighty-five KT recipients from DBD who received basiliximab were included as controls. From KT recipients who received thymoglobulin, 33.6/43.4% presented with an increase in AST/ALT at 72 h post-KT, respectively. Regarding donor type, the percentage of recipients who experienced 72 h post-KT hypertransaminasemia was higher in uDCD group (65.1/83.7% vs. 20.3/26% in DBD and 20.7/27.6% in cDCD, *p* < 0.001). Within the control group, 9.4/12.9% of patients presented with AST/ALT elevation. One month after transplant, AST/ALT values returned to baseline in all groups. The multivariate analysis showed that uDCD recipients had 6- to 12-fold higher risk of developing early post-KT hypertransaminasemia. Early post-KT hypertransaminasemia is a frequent and transient event related to the kidney donor type, being more frequent in uDCD recipients.

## 1. Introduction

Transaminases (alanine aminotransferase (ALT) and aspartate aminotransferase (AST)) are intracellular liver enzymes that act as markers of liver injury, as their elevation usually reflects hepatic cell damage. Other than in the hepatocytes, AST can be found in lower concentrations in the heart muscle, kidneys, and brain, amongst other organs [1]. The normal AST/ALT blood concentration varies depending on laboratory criteria, although normal values range between 30 and 40 IU/L. Both AST and ALT blood concentrations are elevated when the hepatocyte cell membrane is injured. In the general population, the most common causes include alcohol intake, viral hepatitis, fatty liver disease, autoimmune hepatitis, and pharmacological factors [1].

In kidney transplant (KT) recipients, hypertransaminasemia is a frequent finding, affecting 7 to 24% of patients depending on the series [2,3,4,5]. The main causes during the first months after transplant are attributed to viral infections (hepatitis C virus, hepatitis B virus, cytomegalovirus, and Epstein Barr virus), previous liver disease (hepatorenal polycystic disease), and pharmacological hepatotoxicity (immunosuppressants, antibiotics, antifungals and antiviral agents) [2,4,6,7]. Regarding early hypertransaminasemia in the immediate post-transplant period, hemodynamic alterations during the surgical procedure could play a role [7]. In addition, pharmacological hepatotoxicity from immunosuppressants used for induction treatment, specifically thymoglobulin, has been described [8,9,10]. Hypertransaminasemia after thymoglobulin administration has been reported in up to 26% of the patients, especially when high doses are used in the setting of allogeneic stem cell transplantation [8,9,10].

However, these might not be the only reasons behind post-transplant hypertransaminasemia. Interconnection between organs is required for the proper functioning of the organism. The term organ crosstalk refers to the complex interactions maintaining the organism’s homeostasis. In case of organ dysfunction, this crosstalk may also facilitate the dysfunction of other organs through neuronal, endocrine, and paracrine pathways [11,12,13]. Acute kidney injury implies a renal dysfunction where the lesion may not only occur in the kidney, but it also has repercussions in other organs such as the heart, lungs, liver, and bowel [13,14]. There is, therefore, a crosstalk between liver and kidney and, although acute hepatic failure is rarely a consequence of another organ injury, alterations in other organs such as the kidney can affect the liver function [11,15]. The mechanisms of the liver-kidney crosstalk are complex and not fully studied. Systemic inflammation with activation of cytokines, oxidative stress and activation of pro-apoptotic pathways seems to be especially relevant [16,17].

In KT, there is an initial injury to the kidney graft caused by ischemia-reperfusion damage, which results from an initial restriction of blood supply to the graft followed by its restoration and subsequent re-oxygenation. Paradoxically, the restoration of blood flow is often associated with an exacerbation of tissue damage and an inflammatory response activation that causes the so-called reperfusion damage [18]. This initial kidney injury and the kidney–liver crosstalk may cause hepatic damage and might be responsible for post-KT hypertransaminasemia occurrence [11,13]. In that case, the severity of ischemia-reperfusion injury could imply a more severe hepatic damage. Although several factors such as cold ischemia time [19,20,21] have been related to ischemia-reperfusion damage, no studies have analyzed their potential influence on liver function alteration after transplant. In fact, donation after circulatory death (DCD)—especially uncontrolled DCD (uDCD)- confers greater ischemia damage to the organs than donation after brain death (DBD), as it begins with a period of warm ischemia time that may be up to 2–3 h [22]. However, it is unknown if the donor type and its subsequent ischemia-reperfusion damage severity might have a role in post-KT hypertransaminasemia occurrence as a consequence of the kidney–liver crosstalk.

Therefore, the aim of our study was to evaluate the prevalence and evolution of hypertransaminasemia in the immediate post-KT period, if it has any relation with the donor type, and its possible association with post-KT outcomes as a prolonged delayed graft function (DGF).

## 2. Materials and Methods

### 2.1. Study Design and Patients

**Figure 1:**
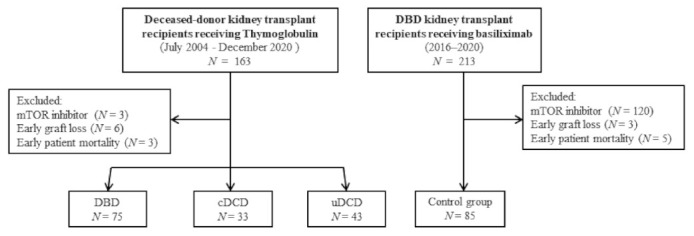
Patients’ flow-chart. DBD: brain death donors, cDCD: controlled after circulatory death donors, uDCD: uncontrolled after circulatory death donors.

This is a retrospective patient cohort study including deceased-donor KT recipients who underwent KT between July 2004 and December 2020 at the Hospital del Mar, Barcelona, Spain. Initially, and to avoid potential confounders, only those recipients who received induction immunosuppression with thymoglobulin were selected (*n* = 163). Patients who received maintenance immunosuppression with mammalian target of rapamycin (mTOR) inhibitor instead of mycophenolic acid (*n* = 3), patients with immediate graft loss (*n* = 6), and those who died in the early post-transplant period (*n* = 3) were excluded from the analysis. A total 151 patients were analyzed, and of those: 43 recipients from uDCD who receive induction with thymoglobulin by protocol in our center; 33 KT recipients from controlled DCD (cDCD); and 75 KT from DBD -who receive thymoglobulin for high immunological risk-. Maintenance immunosuppression consisted of tacrolimus, mycophenolic acid, and prednisone. To be noted, tacrolimus had a delay-start among uDCD KT recipients, initiated between 3 and 5 days after transplant. Then, we added a control group of 85 DBD KT recipients (2016–2020) who received immunosuppression induction with basiliximab and maintenance with tacrolimus, mycophenolic acid, and prednisone. Figure 1 shows the patient’s flow-chart. Patients were followed-up for 3 months after transplantation.

### 2.2. Definitions

Liver alterations detected by image were defined as: liver cysts, liver steatosis and hepatic hemangiomas or angiomyolipomas. Hypertransaminasemia was defined in a qualitative way, as the elevation of ALT/AST above the upper limit of normality in our laboratory (32/33 IU/L, respectively), and quantitatively with the numerical value of AST and ALT (IU/L). Liver function was evaluated through bilirubin elevation above the upper limit of normality in our laboratory (1.20 mg/dL). Unfortunately, other data regarding liver failure as albumin levels and prothrombin time were not available during the early post-transplant period (first 72 h). Regarding types of donors: DBD refers to donors with diagnosis of death based on neurological criteria (severe and irreversible brain injury leading to death); DCD refers to organ donation after confirmation of cessation of circulation, and it can be controlled (cDCD), when cessation of circulation is expected, or uncontrolled (uDCD), when unexpected [23]. DGF was defined as the need of at least one dialysis session within the first 7 days after KT and prolonged delayed graft function in the absence of kidney graft function (creatinine decrease) lasting 14 days or more.

### 2.3. Variables

Clinical data were collected from our local transplant database, including baseline demographic characteristics from donors and recipients, transplant characteristics and clinical follow-up variables periodically registered.

To rule out a possible inadvertent basal liver disease in KT recipients, liver abnormalities identified by ultrasound or abdominal tomography performed during pre-transplant study were gathered. Additionally, other variables with a potential effect on transaminases were collected, such as cold ischemia time as a factor associated with the severity of ischemia-reperfusion damage; transfusion need and use of vasoactive drugs within the first 6 h after transplant (defined as post-KT low BP) as indicators of systemic hypoperfusion; and thymoglobulin total dose (mg/kg) and tacrolimus total dose (mg/kg) administered during the first week post-KT, as well as tacrolimus levels at 7 days post-KT as indicators of possible confounders, and hepatotoxic drugs. No other potential drugs able to induce liver injury were collected, as main drugs that recipients use during the first 72h after transplant are immunosuppressants.

Kidney (serum creatinine) and liver function (transaminases and bilirubin) were evaluated at five time-points during the first 3 months after transplantation: baseline (pre-transplantation), at 72 h post-KT, 7, 30, and 90 days post-KT.

### 2.4. Statistical Analysis

Normality tests were performed in all quantitative variables: in case of meeting normality criteria, results are expressed as mean and standard deviation (SD); otherwise, as median and interquartile range (IQR). Categorical variables are described as counts and percentages. Comparisons between the four groups were conducted using ANOVA test for the variables with normal distribution and Kruskal-Wallis H test for the non-parametric ones. Mann-Whitney U test was used for comparisons between two groups in non-parametric variables. For categorical variables, Chi-Square and McNemar tests were used for the non-related and related groups, respectively.

Two multivariable analyses were also performed using binary logistic regression. The first one to determine factors associated with early hypertransaminasemia after transplant. In the second analysis, our aim was to establish predictors of prolonged delayed graft function among uDCD KT recipients. For adjustment, only those variables with a *p*-value < 0.1 in the univariate analysis were included. Results are expressed as Odds Ratio (OR) and 95% confidence interval (CI). A *p*-value < 0.05 was considered statistically significant. Statistical analysis was performed using SPSS V 20 (SPSS Inc., Chicago, IL, USA).

## 3. Results

Baseline characteristics of the four groups of patients are shown in Table 1. Among uDCD group, both donors and recipients were younger and with a higher percentage of men. In addition, fewer recipients were retransplants in this group. No differences in past medical liver disease (hepatitis B or C virus serology) or hepatic disorders detected by image were seen between the four groups.

Transplant characteristics are shown in Table 2. Cold ischemia time was shorter in the cDCD group when compared with the other three groups (12.6 h vs. 15.6 in DBD-KT, 16.42 in uDCD-KT and 15.3 in the control group). Total accumulated dose of thymoglobulin was also similar among the three main groups. Tacrolimus dose and levels were initially lower in the uDCD group, but no differences were found at 30 days after KT, Table 2.

Regarding renal function evolution, recipients from uDCD presented longer DGF (creatinine decline among uDCD-KT occurred by 16 days after transplantation vs. 2 days in the control group). One week after transplant, and considering those recipients with functioning graft, uDCD-KT presented with poorer kidney function, i.e., serum creatinine 6.2 mg/dl vs. 2.6mg/dl among DBD-KT. However, by three months after transplantation graft function was similar among groups, Table 2.

**Figure 2:**
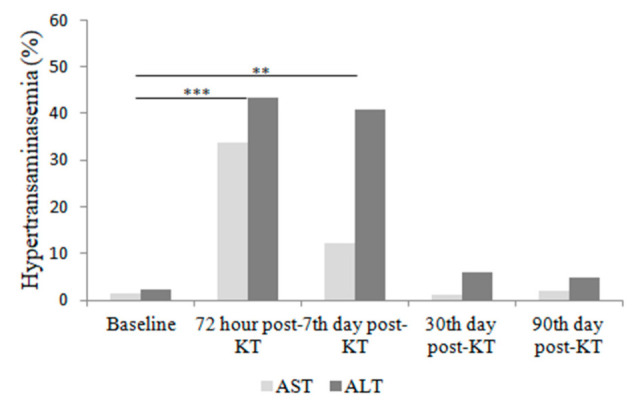
Percentage of patients with elevated transaminases above normal values at different time-points related to transplantation. Comparisons to baseline were made with McNemar test. Differences were found in AST and ALT levels at 72 h and 7 days post-KT. ** *p* < 0.01; *** *p* < 0.001.

When analyzing the three main study groups (DBD, cDCD, uDCD), in terms of hypertransaminasemia and liver function, 36.6 and 43.4% of recipients presented with AST and ALT above normality limits at 72 h post-KT, Figure 2, although this alteration was transient and partially resolved by 7 days after transplant (AST), and completely by 30 days after transplant. No significant elevation of bilirubin was observed at any point (data not shown).

**Figure 3:**
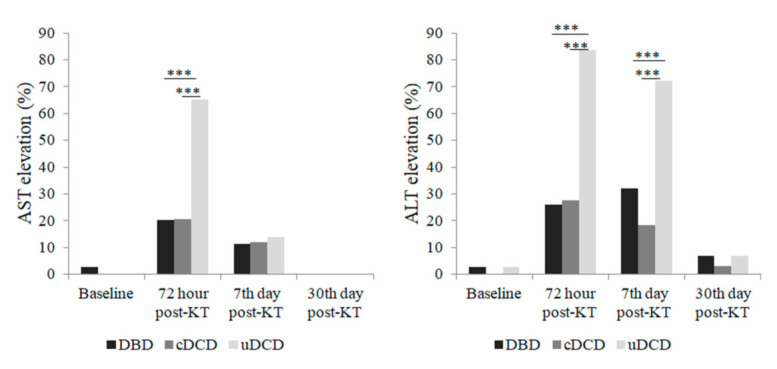
Percentage of patients with elevated AST or ALT according to donor type (only those recipients who received thymoglobulin). Comparisons between groups were made with Chi-Square test. Differences were found in the uDCD group at 72 h post-KT (AST levels) and at 72 h and 7 days post-KT (ALT levels). *** *p* < 0.001.

**Figure 4:**
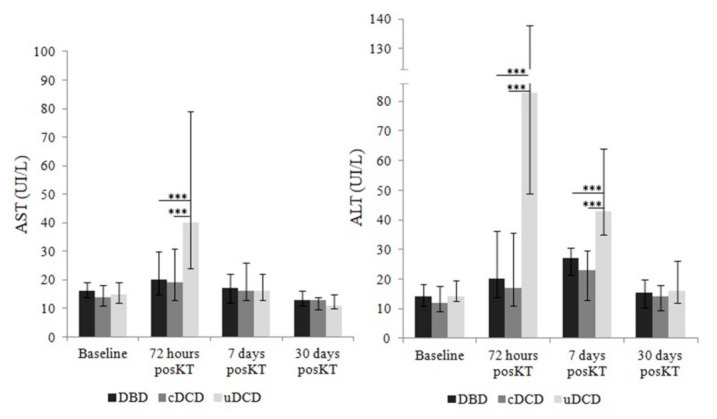
Dimension of AST and ALT elevation according to donor type (only those recipients who received thymoglobulin) represented as medians and interquartile range. Comparisons between groups were made with Mann-Whitney U test. Differences were found in the uDCD group at 72 h post-KT (AST levels) and at 72 h and 7 days post-KT (ALT levels). *** *p* < 0.001.

We analyzed if donor type had any influence in early post-transplant hypertransaminasemia occurrence. The elevation of AST/ALT at 72 h post-KT was much more frequent among uDCD recipients (Figure 3), presenting with elevated AST/ALT 65.1/83.7% in comparison with 20.7/27.6% in cDCD-KT, and 20.3/26% in DBD group. Among KT recipients in the control group, only 9.4/12.9% presented with elevated AST/ALT, respectively. AST and ALT behaved different along time between groups (Figure 3), and these data were confirmed when considering hypertransaminasemia in a quantitative way (Figure 4).

**Figure 5:**
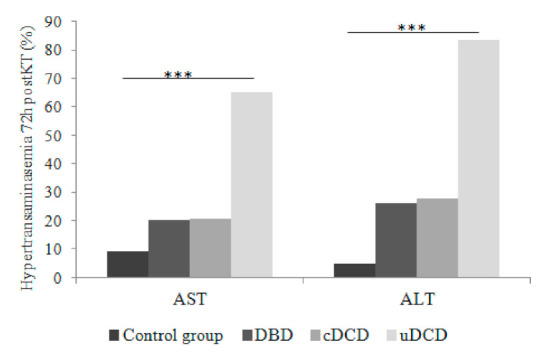
Percentage of KT recipients presenting hypertransaminasemia during the early posttransplant period according to the donor type. Comparisons with the control group. Comparisons between groups were made with Chi-Square test. Differences were found only between the control group and the uDCD group for both AST and ALT. *** *p* < 0.001.

We compared each group of KT recipients who received thymoglobulin with the control group. Figure 5 displays the percentage of recipients presenting with hypertransaminasemia in the early posttransplant period, showing an incremental risk and starting in the control group < DBD-thymoglobulin KT recipients < cDCD-thymoglobulin KT recipients < uDCD-thymoglobulin KT recipients. Despite a lower number of recipients presented hypertransaminasemia within the control group, we found no significant differences when comparing with DBD-thymoglobulin and cDCD-thymoglobulin recipients, but there were significant differences when comparing with uDCD KT recipients.

In the multivariable analysis of predictors for early hypertransaminasemia after transplant, uDCD KT recipients had the highest odds (OR 6.840 for AST elevation and 12.381 for ALT elevation). Cold ischemia time (OR 1.1 per hour) was also associated with AST elevation after transplant. We did not find that thymoglobulin use was independently associated with early hypertransaminasemia (Table 3). On the other hand, a multivariable analysis of predictors for prolonged delayed graft function in uDCD KT recipients was conducted (donor and recipient age and sex, cold ischemia time, post-KT low blood pressure, or AST/ALT elevation within 72h post-KT) and no associations were found with early hypertransaminasemia.

## 4. Discussion

The present study evaluates the prevalence of hypertransaminasemia in the immediate post-KT period, finding that 36.6 and 43.4% of KT recipients who receive thymoglobulin induction suffer AST and ALT elevation, respectively. This elevation occurs early, with a peak at 72 h post-KT, and is followed by an early decline and complete normalization. KT recipients from uDCD are 5 to 13 times more inclined to elevate transaminases than cDCD or DBD ones.

Previous studies have shown that 7–23.3% of KT recipients may present post-transplant hepatic dysfunction but they usually referred to persistent or chronic liver damage (>6 months post-KT) [2,3]. Early hypertransaminasemia after transplant has not been assessed in recent studies. Einollahi et al. [5] analyzed hypertransaminasemia occurrence during the first year after transplantation in 1589 KT recipients from Iran. Out of 110 deceased donor KT recipients, 10.4/23.8% presented with elevated AST/ALT during the first three months after transplant, although the exact moment of appearance and their later evolution is not detailed. Authors found that hypertransaminasemia was more frequent in deceased donor KT recipients than in living donor ones. In this study, we describe a more accurate insight into the liver function alteration early after KT, not previously described in the literature. A high proportion of our KT recipients presented with early hypertransaminasemia (72 h) after transplant, but it was transient, and levels returned to normal values between 7 days and 30 days after transplant.

The association between KT and hypertransaminasemia occurring in the immediate post-KT period might be explained by the liver-kidney crosstalk. During KT, ischemia-reperfusion damage occurs in the kidney graft. Evidence suggests that the inflammatory and oxidative stress associated to this damage may have detrimental effects on the liver [16]. Multiple research studies have described signaling pathways between both organs which begin with the formation of reactive oxygen species during renal reperfusion and ultimately result in the activation of inflammation and cell damage pathways [17]. In a murine model of renal transplantation, Zhao et al. [24] showed that ischemia-reperfusion damage of renal grafts was associated with liver dysfunction. Liver damage was related to significant inflammatory infiltration and increased expression of Toll-like receptor-4 (TLR-4) by histones. Other studies have described the role of other inflammatory mediators such as tumor necrosis factor-alfa [25] and interleukin 17A and interleukin 6 [26].

If ischemia-reperfusion damage is linked to the liver-kidney crosstalk, its severity should correlate to liver damage. Krüger et al. [27] identified the signaling pathway through TLR-4 as key for ischemic reperfusion damage in renal transplantation and found relevant differences in its expression when comparing living and deceased donors. There might be differences in signaling pathways activated during the ischemia-reperfusion damage in the different types of deceased kidney donors. The mechanisms that cause ischemia-reperfusion damage are similar, but there are differences depending on the circumstance. For example, a cardiorespiratory arrest or a shock leads to generalized systemic hypoperfusion while the ischemia produced in an acute myocardial infarction is restricted to the heart [18]. Our study analyzed the influence of donor type in early post-KT hypertransaminasemia. We found that post-KT hypertransaminasemia was more frequent and of a greater magnitude among recipients from uDCD. The multivariable analysis confirmed these data, and KT recipients from uDCD have a higher risk of developing early post-KT hypertransaminasemia. To our knowledge, this is the first study exploring the influence of the deceased donor type on post-KT hypertransminaemia.

Furthermore, to better assess the potential role of thymoglobulin in early hypertransminaemia, we included a control group of 85 recipients from DBD who received basiliximab. We did not find any difference among the control group and DBD-thymoglobulin KT recipients’ group, and thymoglobulin was not associated with early hypertransaminasemia in the multivariable analysis. This reinforces the hypothesis that despite thymoglobulin has been associated with liver toxicity [8,9,10], other mechanisms are displayed early after KT that might be responsible for this liver alteration.

Finally, as early hypertransaminasemia resolved quickly and it might reflect ischemia-reperfusion damage severity [20,28], we aimed to analyze if it could be related to DGF severity. However, we did not find any correlation between hypertransaminasemia and prolonged DGF in uDCD recipients in our cohort.

The limitations of our study are those derived of its single-center and retrospective condition. The low number of uDCD recipients could have reduced the statistical power of the multivariable analysis. However, to our knowledge, this is the first study that explores early post-KT hypertransaminasemia and its relationship with donor type and it can be of interest to the transplant clinician: (1) hypertransaminasemia can be expected more frequently if the donor is uDCD; (2) it is going to be transient; and (3) it could correlate with the severity of I/R injury, although we were not able to demonstrate any association with prolonged DGF.

We conclude that early post-KT hypertransaminasemia is a frequent and transient event related to donor type and being more frequent in recipients from uncontrolled circulatory death donors. More studies are needed to corroborate these results and possibly correlate this finding with the severity of ischemia-reperfusion damage.

## Figures and Tables

**Table 1:** Baseline characteristics of donors and recipients. Comparisons between groups were made with ANOVA or Kruskal-Wallis H tests according to its normal or non-parametric distribution (quantitative variables), and with Chi-Square test (categorical variables).

	DBD (*n* = 75)	cDCD (*n* = 33)	uDCD (*n* = 43)	Control Group (*n* = 85)	*p*-Value
**Donor Characteristics**					
Age (years, mean ± SD)	54.1 ± 15.5	67.6 ± 11.26	49.93 ± 10.96	58.6 ± 21.3	<0.001
Sex (male, *n*, %)	42 (56.8)	21 (63.6)	38 (88.4)	50 (58.8)	0.003
BMI (Kg/m2, median, IQR)	26.4 (24.4–29.4)	25.4 (22.8–29.9)	26.7 (24.2–29.4)	26.8 (24.4–30.8)	0.630
Hypertension (*n*, %)	37 (52.1)	18 (56.2)	13 (31.7)	45(52.9)	0.095
DM (*n*, %)	11 (15.1)	11 (34.3)	3 (7.3)	13 (15.3)	0.017
**Recipient Characteristics**					
Age (years, mean ± SD)	56.2 ± 12.6	63.5 ± 10.9	52.4 ± 10.2	58.1 ± 12.9	0.002
Sex (male, *n*, %)	25 (33.3)	16 (48.5)	31 (72.1)	63 (74.1)	<0.001
BMI (Kg/m2, median, IQR)	25.1 (21.6–30.4)	26.5 (23.7–29.1)	24.8 (20.6–29.1)	26.2 (23.6–31.1)	0.108
DM (*n*, %)	10 (13.3)	13 (39.4)	9 (20.9)	23 (27.1)	0.021
Hypertension (*n*, %)	67 (89.3)	31 (93.9)	39 (90.7)	77 (90.6)	0.902
Retransplantation (*n*, %)	44 (58.7)	11 (33.3)	2 (4.7)	3 (3.5)	<0.001
Liver alterations detected by image (*n*, %)	27 (38)	10 (30.3)	12 (27.7)	23 (27.7)	0.543
Hepatitis B or C (*n*, %)	6 (8)	2 (4.7)	7 (8.2)	7 (8.2)	0.062

DBD: brain death donors, cDCD: controlled circulatory death donors, uDCD: uncontrolled circulatory death donors, BMI: body mass index, DM: diabetes mellitus, SD: standard deviation, IQR: interquartile range.

**Table 2:** Kidney transplant procedure features. Comparisons between groups were made with ANOVA or Kruskal-Wallis H tests according to its normal or non-parametric distribution (quantitative variables), and with Chi-Square test (categorical variables).

	DBD (*n* = 75)	cDCD (*n* = 33)	uDCD (*n* = 43)	Control Group (*n* = 85)	*p*-Value
**Intra and Early Post-Transplant Period**					
Cold ischemia time (hours, mean ± SD)	15.6 ± 5	12.6 ± 5	16.2 ± 5.1	15.3 ± 5.4	0.018
Blood transfusion (yes, *n*, %)	41 (54.7)	20 (60.6)	25 (58.1)	22 (25.9)	<0.001
Post-KT low BP (yes, *n*, %)	16 (23.2)	10 (30.3)	6 (15)	19 (22.4)	0.482
**Initial Immunosuppression**					
Thymoglobulin (mg/kg, median, IQR)	4.4 (3.8–5.3)	4.1 (3–5)	4.5 (3.1–5.1)	---	0.613
Tacrolimus total dose at 7 days (mg/kg, median, IQR)	0.89 (0.68–1.08)	0.83 (0.71–1.09)	0.36 (0.25–0.54)	0.71 (0.62–0.86)	<0.001
Tacrolimus levels at 7 days post-KT (ng/mL, median, IQR)	9.9 (6.5–12.4)	9.4 (7.75–12.1)	4.9 (3.3–9.25)	8.8 (6.7–13.6)	<0.001
Tacrolimus levels at 30 days post-KT (ng/mL, median, IQR)	9.7 (7.5–12.1)	9.7 (7.8–11.7)	8.9 (7.25–10.5)	9.5 (7.9–11.8)	0.522
**Renal Function Evolution**					
Days to creatinine decline (median, IQR)	5 (1–10)	7 (3–14.5)	16 (8.8–27.3)	2 (1–7)	<0.001
Creatinine at 7 days post-KT (mg/dL, mean ± SD)	2.6 ± 1.7	4.5 ± 3	6.2 ± 2.8	3.9 ± 3.3	0.002
Creatinine at 30 days post-KT (mg/dL, mean ± SD)	1.9 ± 1	2.3 ± 1.2	3 ± 2.1	1.9 ± 1.0	<0.001
Creatinine at 90 days post.KT (mg/dL, mean ± SD)	1.8 ± 1	1.9 ± 0.8	2 ± 0.7	1.8 ± 0.7	0.569

DBD: brain death donors, cDCD: controlled circulatory death donors, uDCD: uncontrolled circulatory death donors, KT: kidney transplant, BP: blood pressure, SD: standard deviation, IQR: interquartile range.

**Table 3:** Univariable and Multivariable analysis showing factors associated with early hypertransaminasemia after transplantation. Analyses were made with binary logistic regression.

	AST Elevation 72 h Post-KT Univariable				AST Elevation 72 h Post-KT Multivariable			
	OR	CI 95%		*p*	OR	CI 95%		*p*
Donor age (years)	1.001	0.984	1.018	0.916				
Donor sex (ref. male)	0.657	0.341	1.226	0.209				
Donor DM	1.286	0.526	3.145	0.582				
Donor HTA	1.348	0.726	2.504	0.345				
Donor type (ref. DBD)								
cDCD	1.497	0.550	4.007	0.430	1.317	0.430	4.031	0.630
uDCD	10.713	4.972	23.085	<0.001	6.840	2.834	16.509	<0.001
Recipient age	1.005	0.982	1.029	0.661				
Recipient sex (ref. male)	1.379	0.755	2.519	0.295				
Recipient DM	0.738	0.373	1.458	0.381				
Retransplantation (ref. no)	0.541	0.253	1.156	0.113				
Thymoglobulin (ref. no)	4.610	2.057	10.328	<0.001	2.086	0.793	5.484	0.136
Cold Ischemia Time (hours)	1.094	1.029	1.163	0.004	1.106	1.030	1.188	0.006
Post-KT low BP (ref. no)	1.338	0.654	2.739	0.425				
Blood transfusion (ref. no)	1.872	1.019	3.436	0.043	1.566	0.754	3.252	0.229
Donor age (years)	0.996	0.980	1.012	0.596				
Donor sex (ref. male)	0.443	0.235	0.834	0.012	0.653	0.310	1.372	0.260
Donor DM	0.422	0.616	3.182	0.422				
Donor HTA	1.530	0.862	2.716	0.146				
Donor type (ref. DBD)								
cDCD	1.549	0.625	3.837	0.344	1.293	0.470	3.361	0.619
uDCD	20.914	8.479	51.584	<0.001	12.381	4.515	33.952	<0.001
Recipient age	0.994	0.973	1.016	0.603				
Recipient sex (ref. male)	1.111	0.634	1.949	0.712				
Recipient DM	0.791	0.417	1.500	0.473				
Retransplantation (ref. no)	0.633	0.324	1.234	0.179				
Thymoglobulin (ref. no)	4.749	2.320	9.724	<0.001	2.179	0.938	5.062	0.070
Cold Ischemia Time (hours)	1.063	1.006	1.122	0.030	1.065	0.996	1.139	0.066
Post-KT low BP (ref. no)	0.565	0.268	1.190	0.133				
Blood transfusion (ref. no)	1.273	0.728	2.224	0.397				

KT: kidney transplant, cDCD: controlled circulatory death donors, uDCD: uncontrolled circulatory death donors, DM: diabetes mellitus, RRT: renal replacement therapy, HD: hemodialysis, BP: blood pressure, OR: odds ratio, CI: confidence interval.

## Data Availability

The data presented in this study are available on request from the corresponding author. The data are not publicly available due to confidentiality restrictions.
